# A randomized, single-blind, single-dose study to assess the pharmacokinetic equivalence of the biosimilar ABP 215 and bevacizumab in healthy Japanese male subjects

**DOI:** 10.1007/s00280-018-3695-4

**Published:** 2018-09-29

**Authors:** Vladimir Hanes, Vincent Chow, Zhiying Pan, Richard Markus

**Affiliations:** Amgen Inc., One Amgen Center Drive, Thousand Oaks, CA 91320 USA

**Keywords:** ABP 215, Bevacizumab, Biosimilar, Pharmacokinetics, Reference product

## Abstract

**Purpose:**

Analytic, pharmacokinetic (PK), and clinical similarity between the biosimilar ABP 215 and bevacizumab has previously been demonstrated in global studies. Here we present a phase 1 study in healthy adult Japanese men.

**Methods:**

This study was a randomized, single-blind, single-dose, parallel-group study comparing PK parameters of ABP 215 versus EU-authorized bevacizumab in healthy Japanese men. Primary endpoints were maximum observed serum concentration (*C*_max_) and area under the serum concentration—time curve from time 0 to infinity (AUC_inf_). Secondary endpoints included AUC from time 0 to time of last quantifiable concentration (AUC_last_), safety, tolerability, and immunogenicity.

**Results:**

Baseline characteristics were similar among study subjects (*n* = 24/group). After a 3-mg/kg intravenous infusion, the geometric means (GMs) of *C*_max,_ AUC_inf_, and AUC_last_ were 71.2 µg/mL, 25,259 µg h/mL, and 22,499.3 µg h/mL, respectively, for ABP 215 and 70.16 µg/mL, 25,801 µg h/mL, and 22,604.6 µg h/mL, respectively, for bevacizumab. The GM ratios (90% confidence interval; CI) for *C*_max,_ AUC_inf_, and AUC_last_ were 1.015 (0.946–1.088), 0.979 (0.914–1.049), and 0.995 (0.941–1.053) for ABP 215 versus bevacizumab. All CIs fell within the prespecified bioequivalence margin (0.80–1.25). Adverse events (AEs) occurred in 2/24 subjects receiving ABP 215 and 1/24 receiving bevacizumab. There were no deaths or AEs leading to study discontinuation; no subject was positive for binding anti-drug antibodies (ADAs).

**Conclusions:**

ABP 215 and bevacizumab showed PK similarity in Japanese men. Safety profiles were comparable between the two groups. The pharmacokinetics in Japanese subjects were consistent with those in a previous global PK equivalence study.

## Introduction

Biosimilars are biologic medicines generated, often by a separate company, to be highly similar to an approved biologic reference product developed by an originator company. These entities are developed and approved based on data that demonstrate the similarity between the proposed biosimilar and the reference product with respect to structure and function, as well as similar clinical pharmacology (pharmacokinetics and pharmacodynamics), clinical safety, immunogenicity, and efficacy [1–3].

Bevacizumab is a recombinant humanized immunoglobulin G1 (IgG1) monoclonal antibody that binds and inhibits vascular endothelial growth factor (VEGF); blockade of VEGF signaling by bevacizumab inhibits angiogenesis and can lead to tumor stasis or necrosis [4, 5]. Bevacizumab improves progression-free and overall survival alone or in combination with other approved cancer therapies [4, 5]. ABP 215 (MVASI™ [bevacizumab-awwb]; Amgen Inc., Thousand Oaks, CA, USA) is the first approved biosimilar to bevacizumab (Avastin^®^) [6, 7]. ABP 215 is approved in the United States (US) and the European Union (EU) for the treatment of metastatic colorectal cancer; non-squamous non-small cell lung cancer; glioblastoma; metastatic renal cell carcinoma; and persistent, recurrent, or metastatic carcinoma of the cervix. In the EU, ABP 215 is also approved for treatment of metastatic breast cancer and recurrent epithelial ovarian, fallopian tube, or primary peritoneal cancer, excluding glioblastoma [7].

A comprehensive analytical characterization has shown that ABP 215 and bevacizumab are physicochemically and functionally similar [8]. Pharmacokinetic (PK) similarity between ABP 215 and bevacizumab was previously demonstrated in a large single-dose phase 1 study that included 202 healthy adult men in the EU and US [9]. Additionally, in a recent global phase 3 study that included 642 patients with advanced non-small cell lung cancer (NSCLC), ABP 215 and bevacizumab were shown to be equivalent with respect to clinical efficacy, safety, immunogenicity, and pharmacokinetics [10].

Here we present results from a phase 1 study demonstrating PK similarity between ABP 215 and EU-authorized bevacizumab reference product (hereafter referred to as bevacizumab) in healthy adult Japanese men.

## Methods

### Ethical conduct of the study

This study was conducted in accordance with the provisions of the Declaration of Helsinki, the US FDA Code of Federal Regulations, the International Conference on Harmonization E6 Guidelines on Good Clinical Practice, and the provisions of the EU Clinical Trial Directives. Written informed consent was obtained from each subject at the screening visit prior to the initiation of any study-related procedures.

### Investigational product

ABP 215 was sourced from Amgen, Inc. (Thousand Oaks, CA, USA). Bevacizumab reference product was sourced from Hoffmann-La Roche, Inc. (Basel, Switzerland). Investigational drugs were supplied in single-use vials with 16 mL of solution containing 400 mg of investigational product (25 mg/mL). All subjects in the ABP 215 group received investigational product (IP) from a single lot.

### Study population

Healthy first- or second-generation Japanese men between 18 and 45 years of age were included in this study. Inclusion criteria were BMI ≥ 18.0 and ≤ 25.0 kg/m^2^ and normal or clinically acceptable physical examination, clinical laboratory test values, vital signs, and cardiac function. Exclusion criteria included men of reproductive potential unwilling to practice a highly effective method of birth control for the duration of the study and continuing 6 months following treatment with IP; men who were unwilling to refrain from donating sperm during the study and for 6 months following treatment with IP; men with pregnant partners; history or evidence of a clinically significant disorder that posed a risk to subject safety or interfered with the study; history of surgery or major trauma within 12 weeks of screening or surgery planned during the study; use of any over-the-counter or prescription medications within 14 days or 5 half-lives (whichever was longer); receiving or had received other investigational drugs (or was using an investigational device) within 30 days or 5 half-lives (whichever was longer) prior to receiving; or prior exposure to bevacizumab or related compounds.

### Study design

**Fig. 1:**
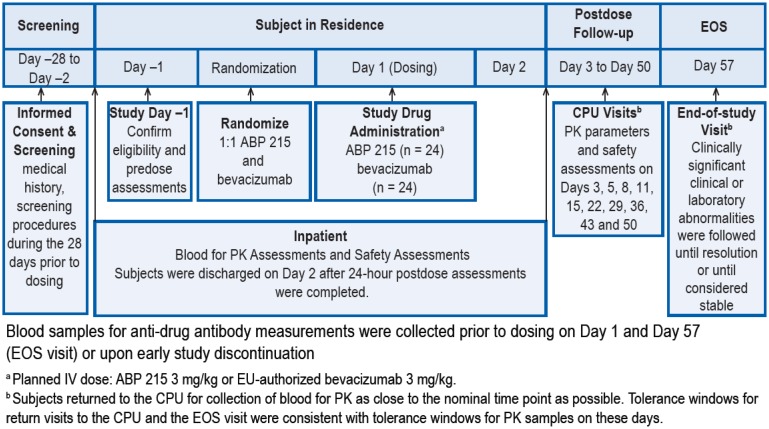
Study design

This randomized, single-blind, single-dose, two-arm, parallel group study compared the PK profiles of ABP 215 and bevacizumab reference product in healthy adult Japanese men (Fig. 1).

Subjects were admitted to the clinical pharmacology unit (CPU) on day − 1 at which time the day − 1 assessments were performed; both the screening and day − 1 results were reviewed to confirm eligibility. Eligible subjects were randomized prior to dosing on day 1 (i.e., on day − 1 or prior to dosing on day 1) according to a computer-generated randomization schedule to receive an intravenous (IV) infusion over 90 min of ABP 215 (3 mg/kg) or EU-authorized bevacizumab (3 mg/kg) in a ratio of 1:1. Subjects were blinded to the treatment; study personnel, including pharmacists, investigators, nurses, sponsor, etc., were unblinded to treatment. Dosing occurred on day 1 after pre-dose baseline procedures were completed. Subjects resided in the CPU for at least 24 h after dosing for safety and PK assessments. The subjects were discharged on day 2 after completion of study procedures. Subjects returned to the CPU on days 3, 5, 8, 11, 15, 22, 29, 36, 43, 50, and 57 [end-of-study (EOS) visit] for safety evaluations and PK assessments.

Subjects received a single 90-min IV infusion of ABP 215 (3 mg/kg) or bevacizumab (3 mg/kg) on the morning on day 1. Subject weight as recorded on day − 1 was used to calculate the actual dose for infusion. In cases in which infusion-related symptoms occurred, the infusion could be temporarily discontinued for up to 30 min or the infusion rate could be lowered based on assessment by the investigator or a delegated physician. The IP was administered by authorized, trained healthcare professionals.

### Study objectives

The primary objective of this study was to demonstrate PK bioequivalence determined by comparing the AUC_inf_ and *C*_max_ in subjects treated with ABP 215 to those treated with bevacizumab. The secondary objective was to assess the safety, tolerability, and immunogenicity of ABP 215 compared with bevacizumab.

### Blood sampling and assays

Serum ABP 215 and bevacizumab concentrations were measured from blood samples that were collected prior to dosing and at the following time points: 1.5 (end of infusion), 4, 8, 12, and 24 h after the start of infusion; each return visit to the CPU (days 3, 5, 8, 11, 15, 22, 29, 36, 43, and 50); and at the EOS visit (day 57). Subjects were monitored throughout the study for AEs, clinical laboratory results, concomitant medication use, and vital signs.

Serum concentrations of ABP 215 and bevacizumab were quantified using a validated electrochemiluminescence (ECL) assay that employs a mouse anti-bevacizumab monoclonal antibody (mAb) to capture the IP. After ABP 215 or bevacizumab capture by the immobilized antibody, unbound materials were removed, and ruthenium-labeled mouse anti-bevacizumab mAb was added to detect the captured ABP 215 or bevacizumab. A tripropylamine buffer was added to enhance the ECL signal. The ECL counts were directly proportional to the amount of ABP 215 or bevacizumab bound by the capture reagent. Conversion of ECL counts to concentrations was performed using Gen5™ Secure Software v1.08.

Binding and neutralizing ADAs were detected using a two-tiered approach that included a screening assay and a confirmatory assay. Sampling for ADAs occurred pre-dose on day 1 and at the EOS visit. A validated immunoassay was used to detect antibodies capable of binding ABP 215 and bevacizumab. Any sample positive for ADA binding was to be assessed for neutralizing antibodies capable of binding to ABP 215 or bevacizumab using a ligand—(VEGF) binding assay.

### Study endpoints

The primary endpoints for this study were the PK parameters AUC_inf_ and *C*_max_. Secondary endpoints were the incidence of treatment emergent adverse events (AEs), vital signs, laboratory safety tests, electrocardiograms (ECGs), incidence of ADAs, and AUC from time 0 to the last quantifiable concentration (AUC_last_).

### Pharmacokinetic evaluation

PK parameters assessed included maximum observed serum concentration (*C*_max_), area under the serum concentration–time curve from time 0 extrapolated to infinity (AUC_inf_), area under the serum concentration–time curve from time 0 to the last quantifiable concentration (AUC_last_), last measurable serum concentration (*C*_last_), time at which *C*_max_ was observed (*t*_max_), terminal elimination half-life (*t*_½_), and first-order rate constant of drug associated with the terminal portion of the curve (*λz*). All values were calculated from serum ABP 215 and bevacizumab concentration data using non-compartmental methods.

### Safety assessments

Subjects were monitored for AEs throughout the study. Vital signs were measured at every CPU visit. Clinical laboratory tests (hematology, chemistry, and urinalysis) were administered at screening and days − 1, 2, 8, 22, 43, and EOS (day 57). Physical examinations and 12-lead electrocardiograms (ECGs) were administered at screening and days − 1, 2, and EOS. ADA assessments were performed at day 1 (pre-dose) and EOS.

### Statistical analysis

The serum concentration versus time profile was summarized and depicted descriptively for all subjects who received any amount of IP and had at least one reported serum concentration of ABP 215 or bevacizumab. PK parameters were calculated using non-compartmental techniques (WinNonlin^®^ Professional Network Edition, Version 6.3, Pharsight Corp, St. Louis, MO) for all subjects with an evaluable ABP 215 or bevacizumab serum concentration versus time profile. The point estimate and 90% confidence intervals (CI) for the ratio of the least square geometric means (GMs) for *C*_max_, AUC_inf_, and AUC_last_ were estimated using an analysis of variance model. PK similarity criteria were prespecified using the standard bioequivalence margin, comparing the 90% CIs for the geometrical mean (GM) test-to-reference ratios for *C*_max_ and AUC_inf_ within 0.80 and 1.25; AUC_last_ was also evaluated to fully assess exposure to the IP. To establish bioequivalence, the 90% CIs for the GM test-to-reference ratios for *C*_max_, AUC_inf_, and AUC_last_ had to be entirely contained within the bioequivalence margin. PK parameters were log-transformed prior to statistical modeling.

The safety population consisted of all subjects who were randomized and received any amount of the IP. AEs were listed by system organ class and preferred term (Medical Dictionary for Regulatory Activities, Version 18.0) and summarized by severity and relationship to treatment. The PK concentration population consisted of all subjects who were randomized and received any amount of IP and had at least one reported serum concentration of ABP 215 or bevacizumab. The PK parameter population consisted of all subjects with an evaluable ABP 215 or bevacizumab serum concentration–time profile to obtain at least one PK parameter. The per protocol PK population consisted of all subjects with an evaluable ABP 215 or bevacizumab serum concentration–time profile who did not experience a key protocol deviation affecting the PK data. The ADA population consisted of all subjects who were randomized, received any amount of IP, and had at least one evaluable ADA test. All analyses were performed according to the IP received. Per protocol, a subject was considered ADA positive if any ADA sample, including the pre-dose sample, was reported positive. A subject was considered ADA negative if all ADA samples were reported negative.

PK equivalence criteria was considered to have been met if the 90% CIs for the ratio of least square GMs of primary PK parameters of ABP 215 versus bevacizumab fell within the bioequivalence criteria of 0.80 and 1.25.

## Results

### Subject disposition and characteristics

Forty-eight subjects enrolled in the study, all of whom completed the infusion. Forty-six (95.8%) subjects completed the study; 2 (4.2%) subjects, both in the bevacizumab group, were lost to follow-up. Overall, age, BMI, and the allocation of first- and second-generation Japanese subjects were similar between the two treatment groups (Table 1).

**Table 1:** Summary of demographic data and baseline characteristics

	ABP 215 (*N* = 24)	Bevacizumab (*N* = 24)
Age, mean (SD), years	32.8 (7.94)	32.5 (6.21)
Race, *n* (%)		
First generation Japanese	19 (79.2)	20 (83.3)
Second generation Japanese	5 (20.8)	4 (16.7)
BMI, mean (SD), kg/m^2^	22.3 (1.62)	21.7 (2.04)

*SD* standard deviation, *BMI* body mass index

### Pharmacokinetic results

**Fig. 2:**
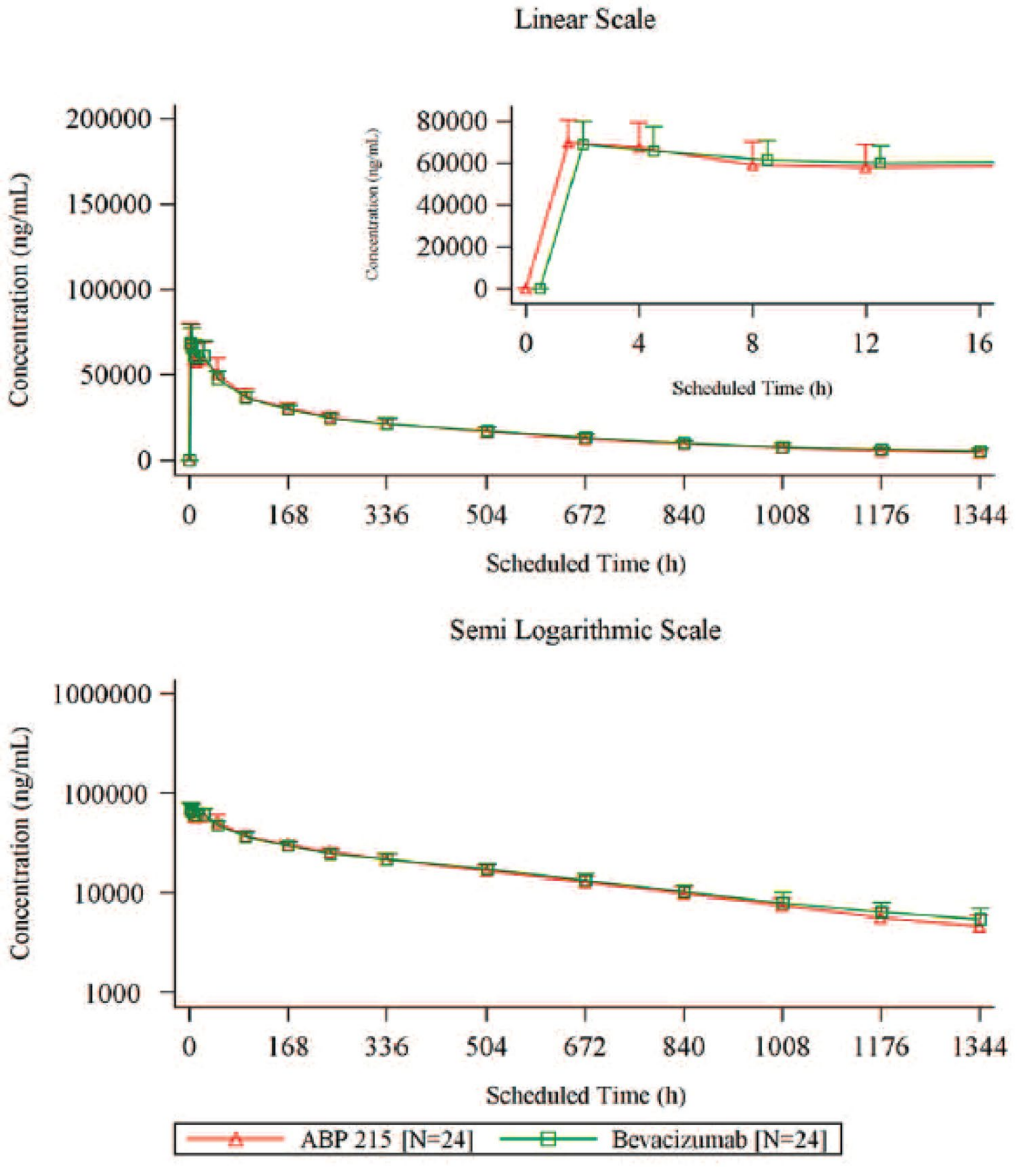
Mean (+ SD) serum ABP 215 and bevacizumab concentration–time profile

Serum concentration–time curves were similar over the course of sampling following a single 3-mg/kg IV infusion of ABP 215 or bevacizumab (Fig. 2). Peak concentrations were observed approximately 2 h after the start of the infusion. In both treatment groups, drug concentrations decreased in a biphasic manner following peak concentration. PK parameters were similar in both groups (Table 2). Peak and overall exposure were similar, as was *t*_max_. Terminal *t*_½_ was estimated to be approximately 18 days. AUC_last_ accounted for at least 90% of the total AUC in the majority of subjects. The bioequivalence assessment of PK parameters for ABP 215 and bevacizumab is shown in Table 3. The 90% CIs for the ratios of geometric means for *C*_max_, AUC_inf_, and AUC_last_ of ABP 215 versus bevacizumab were fully contained within the 0.80–1.25 confidence interval, confirming bioequivalence between ABP 215 and bevacizumab in this population.

**Table 2:** Pharmacokinetic parameters

Treatment	*C*_max_ (µg/mL) GM [*n*] (GeoCV%)	*C*_last_ (µg/mL) GM [*n*] (GeoCV%)	AUC_last_ (µg h/mL) GM [*n*] (GeoCV%)	AUC_inf_ (µg h/mL) GM [*n*] (GeoCV%)	*t*_max_ (h) median [*n*] (min, max)	*t*_1/2_ (h) mean [*n*] (SD)
ABP 215	71.20 [24] (15)	4.42 [24] (30)	22,499 [24] (11)	25,259 [24] (14)	2.84 [24] (1.5, 48.9)	430.2 [24] (60.37)
Bevacizumab	70.16 [24] (14)	5.37 [24] (37)	22,605 [23] (12)	25,801 [21] (14)	1.58 [24] (1.5, 24.0)	470.7 [21] (69.22)

*GeoCV%* geometric mean coefficient of variation

**Table 3:** Statistical assessment of PK parameters

Treatment and comparison	*C*_max_ (µg/mL) LS geometric mean [*n*]	AUC_inf_ (µg h/mL) LS geometric mean [*n*]	AUC_last_ (µg h/mL) LS geometric mean [*n*]
ABP 215	71.20 [24]	25259.1 [24]	22499.3 [24]
Bevacizumab	70.16 [24]	25801.0 [21]	22604.6 [23]
	Ratio of LS geometric means (90% CI)		
ABP 215 versus bevacizumab	1.015 (0.9463, 1.0881)	0.979 (0.9137, 1.0490)	0.995 (0.9410, 1.0528)

*LS* least square

### Safety results

A summary of the AEs is shown in Table 4. Two (8.3%) subjects in the ABP 215 group and one (4.2%) subject in the bevacizumab groups experienced AEs. No subject experienced an infusion reaction or hypersensitivity AE in the first 2 days after study drug administration.

**Table 4:** Summary of adverse events

AE category, *n* (%)	ABP 215 (*N* = 24)	Bevacizumab (*N* = 24)
Subjects with any AE	2 (8.3)	1 (4.2)
Diarrhea	1 (4.2)	0
Malaise^a^	1 (4.2)	0
Nasopharyngitis	0	1 (4.2)
Pyrexia^a^	1 (4.2)	0

Treatment-emergent AEs were defined as all events starting or worsening after commencement of treatment with the IP

*N* number of subjects, % percentage of subjects

^a^Malaise and pyrexia events occurred in the same subject at the same time

No clinically relevant changes in clinical laboratory tests, ECGs, vital signs, or physical examinations were recorded. Pre-existing ADAs were not detected in any of the baseline samples, and no subject had positive binding ADAs at any time point during the study.

## Discussion

Bevacizumab is approved for use in the US, EU, and Japan for the treatment of several types of cancer. Bevacizumab has been shown to improve survival either alone or in combination with other cancer therapies [1–3]. ABP 215 has been shown to be physicochemically and functionally similar to bevacizumab [8]. ABP 215 and bevacizumab also have similar PK profiles in healthy adult males following a single IV dose [9]. More recently, the efficacy, safety, and immunogenicity of ABP 215 and bevacizumab were compared in a phase 3 study in 642 patients with advanced non-small cell lung cancer [10]. In that study, the risk ratio of objective response rate (ORR) and 90% CI between ABP 215 and bevacizumab were within the prespecified equivalence margin, indicating clinically equivalent efficacy between ABP 215 and bevacizumab [10]. Thus, the totality of evidence to date support clinical equivalence of ABP 215 and bevacizumab. In addition, scientific justification for extrapolation across bevacizumab indications was provided to regulatory agencies for approval of all available indications and totality of evidence.

In this study, healthy subjects were enrolled because they are the most homogenous population for a sensitive assessment of the PK similarity without the potential confounding effects of comorbidities or concomitant drug therapy, which can alter PK profiles. Blood sampling through day 57 was sufficient to adequately characterize the ABP 215 and bevacizumab concentration–time profiles; this duration represents more than three times the *t*_1/2_ and provided more than 80% of AUC_inf_ in > 90% of subjects. ADAs were measured to assess immunogenicity, which is a potential risk with all therapeutic proteins.

All biosimilars have minor physicochemical differences compared with their reference product. These differences are attributed to differences in cell lines, as well as the manufacturing and purification processes. Demonstrating similarity with respect to structure and in vitro biologic function and clinical PK is the foundation for establishing biosimilarity, while clinical equivalence in efficacy, safety, and immunogenicity is an important step in confirming clinical equivalence.

The Japanese regulatory authority [the Pharmaceuticals and Medical Devices Agency (PMDA)] published guidelines on biosimilars, known as “follow-on biological products” (FOBPs) in Japan, in 2009 [11–13]. According to the PDMA, a follow-on biological product is a new biotechnological medicinal product developed to be similar to an already licensed, biotechnology medical product. FOBPs are developed on the basis of data that demonstrate structural, functional, pharmacokinetic, and clinical comparability between the FOBP and the reference product. The guidance recommends a single-dose study to assess safety and PK of investigational drugs in healthy Japanese volunteers or patients to compare safety and PK with non-Japanese populations based on the bridging concept described in the International Conference on Harmonisation E5 Guidelines.

In summary, in this study, a single dose of ABP 215 or bevacizumab administered to healthy male Japanese subjects resulted in equivalent PK profiles and safety; no new safety signals with regard to treatment with ABP 215 or bevacizumab were reported. PK equivalence was achieved for the PK endpoints, AUC_inf_, AUC_last_ and *C*_max_, confirming that ABP 215 is similar to bevacizumab in healthy male Japanese subjects. These data are consistent with the PK equivalence previously demonstrated in a single-dose PK study that included 202 healthy adult men in the US and EU and adds to the evidence of ABP 215 as a high quality biosimilar to bevacizumab for use in Japan [9].
